# High-Fidelity, Indigenously Prepared, Low-Cost Moulage as a Valid Simulation Tool to Improve Trauma Education

**DOI:** 10.7759/cureus.57451

**Published:** 2024-04-02

**Authors:** Arun Varghese, Hemanth Kumar, Rajesh Kathrotia, Madhur Uniyal, Shalinee Rao

**Affiliations:** 1 College of Nursing, All India Institute of Medical Sciences, Gorakhpur, Gorakhpur, IND; 2 Advanced Center of Medical Simulation and Skills, All India Institute of Medical Sciences, Rishikesh, Rishikesh, IND; 3 Physiology, All India Institute of Medical Sciences, Rajkot, Rajkot, IND; 4 Trauma and Critical Care, All India Institute of Medical Sciences, Rishikesh, Rishikesh, IND; 5 Pathology, All India Institute of Medical Sciences, Rishikesh, Rishikesh, IND

**Keywords:** suspension of disbelief, injury, simulation in medical education, standardized patient, trauma education, special effects makeup, immersion, realism, fidelity, makeup

## Abstract

Background

Simulation-based trauma education facilitates repeated practice in a controlled and safer environment for the learner without any risk to the patient’s well-being. Moulage contributes to the perception of reality during training using standardized patients. However, the high cost of commercial moulage items is often prohibitive for regular use. This study aimed to assess the effectiveness of indigenously prepared, low-cost moulage as a valid simulation tool to improve trauma education, explore possible replacements of commercial moulage products, and determine their merits and demerits.

Methodology

Readily available economic items were used to make low-cost moulage on the simulated patients to replicate trauma victims. A cross-sectional design used a pre-validated Modified Moulage Authenticity Rating Scale to collect data from 61 participants of Advanced Trauma Life Support and Advanced Trauma Care for Nurses courses to analyze the effectiveness and fidelity of moulage.

Results

In total, 54 (89%) participants scored the low-cost moulage to provide high fidelity effectively. The majority of respondents graded the authenticity of moulage as good. Overall, 46 (75%) participants felt moulage injuries were quite realistic. All agreed that the moulage-based simulation offered a good teaching-learning alternative to assess and manage trauma victims. Further, 45 (73%) participants felt they were in an actual clinical situation, and 58 (95%) stated it could help them in their clinical practice.

Conclusions

Indigenously prepared, low-cost moulage is a feasible and cost-effective means to enhance fidelity in simulation-based trauma education. It can also be a possible replacement for commercial moulage. Further research is needed to rigorously evaluate the effectiveness of indigenously prepared, cost-effective moulage in trauma education to enhance patient care outcomes. This technique can also be easily translated into other simulation-based medical education domains.

## Introduction

Simulation-based medical education is a widely utilized tool for experiential learning to resemble clinical practice among health professionals. Simulation-based trauma education allows the participants to provide repeated practice in a controlled and safe environment [1]. Trauma care is a set of complex critical events that present patient safety at risk. The clinical implementation of simulation-based training can improve patient care in actual trauma resuscitations [2]. It helps to realistically replicate trauma scenarios with the help of standardized patients (trained laypeople). One of the methods to improve fidelity in simulation-based medical education is using moulage [3].

Moulage is the process of applying makeup to increase fidelity in simulation by creating realistic injuries, diseases, aging, and other physical changes to give real-world experience [4]. Although simulation-based education is an effective method for trauma education, it can be costly. The cost can be reduced by various means, such as using low-cost moulage items instead of conventional commercial ones. Although a low-cost moulage is a good option, it can only be effective if it provides high fidelity [5]. The fidelity of the moulage is the level of closeness to real-life injuries or clinical conditions and plays a crucial role in suspending disbelief in simulated scenarios. Suspension of disbelief refers to the temporary acceptance of the situation to be real and allowing the individuals to engage in an immersive learning environment.

Hence, the level of fidelity of the low-cost moulage needs to be high and evaluated to determine its authenticity and effectiveness. This study aimed to assess the effectiveness of indigenously prepared, low-cost moulage as a valid simulation tool to improve trauma education, explore the possible replacements of commercial moulage products, and determine their merits and demerits.

Previous studies have described low-cost moulage techniques, their advantages, and how they can mimic real wounds [4-6]. However, none of these studies compared low-cost moulage with commercial moulage in terms of costs, availability in the market, merits, and demerits. Furthermore, this study may be one of the few to consider readily available cheaper alternatives to commercial moulage items without compromising their fidelity.

The abstract of this paper was previously presented at the virtual Medical Education Conference (MEDUCON-2021) organized by the Department of Medical Education, Jawaharlal Institute of Postgraduate Medical Education & Research (JIPMER), Puducherry, in association with the Alumni Association of NTTC JIPMER (AAND) on September 3, 2021.

## Materials and methods

This study followed a cross-sectional descriptive design to assess the effectiveness of low-cost moulage as a valid simulation tool to improve trauma education. A purposive sampling technique was used to recruit 61 participants among 286 Advanced Trauma Life Support (ATLS)/Advanced Trauma Care for Nurses (ATCN) course participants who were either instructors (faculty) or students. All the ATLS participants were doctors, and all the ATCN participants were nurses with experience in treating trauma and critical care patients in real hospital settings. The Modified Moulage Authenticity Rating Scale (MMARS) was sent to the participants as Google Forms via email, to which 61 participants responded and consented to participate in the study. This study was conducted in the context of ATLS and ATCN courses that utilized standardized patients to simulate trauma case scenarios.

Standardized patients were recruited after informed consent for participation in the study and permission to publish audio-visual materials. The course instructor (faculty) explained and trained each of them about the scenario and how to act as a real patient to simulate a set of signs and symptoms. The first and second authors, trained in the basics of makeup and moulage by a film expert, applied moulage on each simulated patient according to the scenario the ATLS/ATCN instructors provided. Four skill stations were set up to mimic the intensive care unit set-up, each lasting an hour. All participants were required to rotate through all four skill stations, and they managed the simulated patients according to the ATLS protocol.

Commercial moulage

Various commercial moulage supplies are available in the market, ranging from professional Special FX kits, which include everything needed to create special effect makeups, to readily available moulage skins that can be applied on the simulated patient or manikin with minimal makeup. In this study, the ready-made skins were not used for comparison as the researchers found them exponentially costly and not readily available. The researcher purchased a few commercial moulage items listed in Table 1 from online shopping sites such as Flipkart and Amazon, as they were unavailable in the local market. The items mentioned in Table 1 are the prices for the minimum purchase quantity of items that can be readily purchased and which are of the best quality available in the market.

**Table 1:** Comparison of the approximate cost of items used in commercial moulage and the possible low-cost replacements. Common items used: basic makeup set with an all-size brush, moulage spatula, foundation powder, skin color agents, makeup sponge, hairdryer, soap and water, towel, etc. *: The cost and quantity mentioned are the prices for the minimum purchase quantity of items that can be readily purchased and which are of the best quality (author’s subjective experience) available in the market; ₹: Indian rupee.

Commercial moulage items*			Low-cost moulage replacements*	
Serial number	Items	Approximate cost	Items	Approximate cost
1	Wax mould 100 g, Liquid latex 500 mL	3,400 ₹, 2,700 ₹	Cornflour power 100 g - 20 ₹, Gelatine powder 50 g - 40 ₹, Liquid paraffin 100 mL - 40 ₹, Glycerine 100 mL - 35 ₹, vaseline 100 g - 120 ₹, pinch of skin color agent	To make moulage skin of 100 g costs 120 ₹
2	Fake tears and sweat liquid 58 mL	744 ₹	Glycerine 100 mL + water	To make 100 mL costs 35 ₹
3	Artificial arterial blood 473 mL	4,500 ₹	Cornstarch syrup 250 mL - 300 ₹, gelatine powder 50 g - 40 ₹, glycerine 100 mL 35 ₹, coco-powder 250 g - 200 ₹, color agents (each color contains 30 mL) - 500 ₹	To make 250 mL costs 210 ₹
4	Artificial blood clot 50 g	3,000 ₹	Cornstarch syrup 250 mL - 300 ₹, gelatine powder 50 g - 40 ₹, glycerine 100 mL - 35 ₹, coco-powder 250 g - 200 ₹, color agents (each color contains 30 mL) - 500 ₹	To make 200 g costs 250 ₹

Low-cost moulage

**Figure 1:**
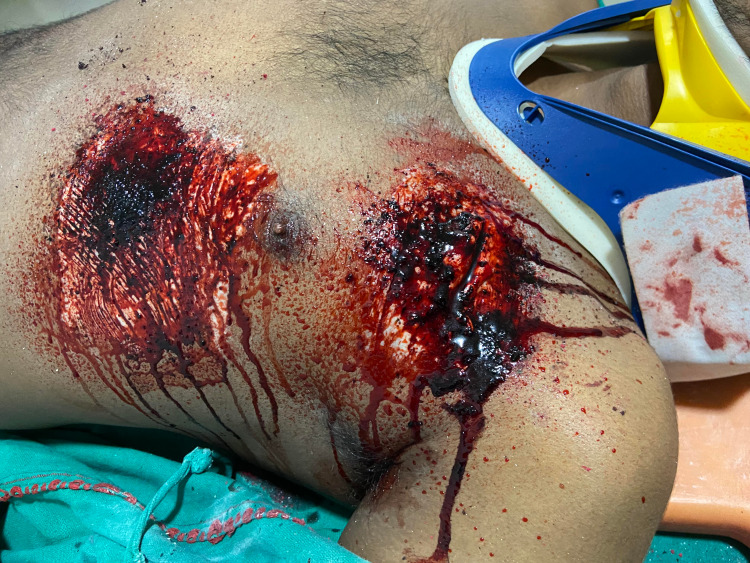
Example of low-cost moulage to simulate chest injury.

**Figure 2:**
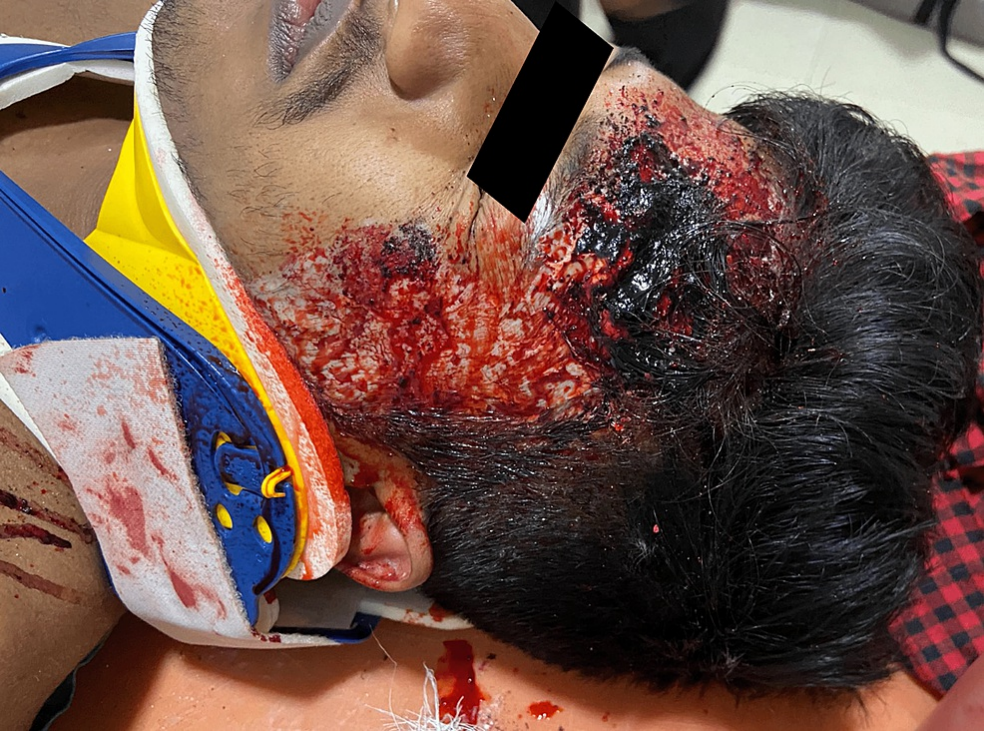
Example of low-cost moulage to simulate head injury.

**Figure 3:**
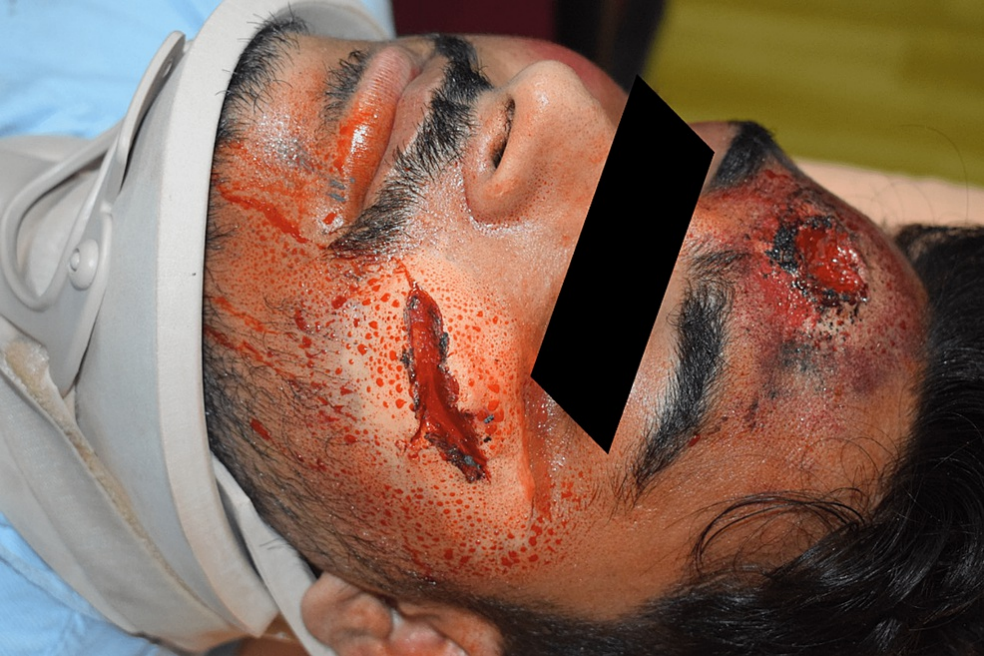
Example of low-cost moulage to simulate gunshot injury and facial laceration.

The first and second authors searched for possible replacements in place of commercial moulage items. After a few trials and errors, they found a few possible replacements commonly used in our day-to-day life, which could be purchased as per the need in small quantities in the local market and which were also economical (Table 1). The researchers tried to identify possible low-cost alternatives for the moulage as they found the conventional moulage materials too costly (Table 2). All moulages were done on standardized patients in this study. Standardized patients were laypeople who agreed to act as patients and were trained to act as per the scenarios given to them (Figures 1-3).

**Table 2:** Comparison of the approximate cost per unit of moulage made in ATLS/ATCN course using commercial moulage items and low-cost moulage replacements. ATLS = Advanced Trauma Life Support; ATCN = Advanced Trauma Care for Nurses

List of moulage	Commercial moulage items	Low-cost moulage replacements
Gunshot injury	250 ₹	20 ₹
Laceration, abrasion, and Bruise	250 ₹	30 ₹
Diaphoresis	100 ₹	15 ₹
Neck vein distension	100 ₹	20 ₹
Stab wound	400 ₹	60 ₹
Active bleeding	450 ₹	70 ₹
Open fracture	1,000 ₹	150 ₹

Modified Moulage Authenticity Rating Scale

The Moulage Authenticity Rating Scale (MARS) consists of 13 indicators for moulage authenticity and is scored on a three-point rating scale, namely, low authenticity, mid authenticity, and high authenticity, and was scored as 0, 1, and 2, respectively. The higher the score, the higher the authenticity [7].

The MARS was modified according to the needs of the study, after permission from the author to check the effectiveness of the moulage. MMARS was formulated based on moulage quality indicators derived from MARS and adding a few more components as appropriate after getting face and content validity from two ATLS, two ATCN, and two simulation experts. MMARS consists of 15 questions to assess the effectiveness and fidelity level of moulage, scored on a three-point rating scale, with an open-ended question for suggestions to improve low-cost moulage. The higher the total score, the higher the fidelity and effectiveness of moulage.

The data were collected via Google Forms and compiled and analyzed using Microsoft Excel to derive the results. Before the commencement of the study, the Institutional Ethical Committee (reference number: 402/IEC/IM/NF/2019) gave ethical approval. All participants were exposed to commercial and low-cost moulage on various occasions (courses) in the simulation lab other than the ATLS/ATCN course. The confidentiality and anonymity of participants were ensured throughout the study.

## Results

A total of 61 participants responded to the MMARS, distributed through Google Forms, the same day after the training. Among these, 12 were faculty and 49 student participants. Of the 61 responses, 24 were ATLS participants, and 37 were ATCN participants.

The majority of respondents graded the authenticity of moulage as average to good. Overall, 46 (75%) participants felt injuries depicted by moulage were quite realistic, while 15 (25%) felt they were somewhat realistic. All agreed that this moulage-based simulation offered a good teaching-learning opportunity to assess and manage trauma victims. In total, 45 (74%) participants agreed that the appearance of the moulage actor made them feel like they were in a real situation. Further, 35 (57%) opined that this experience was better than previous experiences with moulage-based simulation (Table 3). Of the participants, 54 (89%) scored low-cost moulage to have high fidelity (Table 4). Almost all participants in our study indicated that using low moulage is a means of enhancing trauma education. Suggestions from the responders for improving low-cost moulage were training, tutorials, or workshops on moulage from experts that can help improve moulage fidelity. Collaboration with local artists or special effects professionals. Developing standardized guidelines for moulage preparation. Keep an album of real case scenarios Photos as a reference for making each type of moulage. Use of artificial intelligence tools in formulating referral photos for moulage. Moulages made during training can be preserved to save time in future training if possible.

**Table 3:** Participants’ response on the Modified Moulage Authenticity Rating Scale (MMARS) regarding indigenously prepared low-cost moulage in the simulation during ATLS/ATCN training (N = 61). ATLS = Advanced Trauma Life Support; ATCN = Advanced Trauma Care for Nurses

Serial number	Topics			
	Authenticity	Good, f (%)	Average, f (%)	Not optimum, f (%)
1	Authenticity of the color of injuries	53 (87)	8 (13)	0
2	Authenticity of the size of injuries	52 (85)	9 (15)	0
3	Authenticity of the anatomic location of injuries	57 (93)	4 (07)	0
4	Authenticity of the shape of injuries	50 (82)	11 (18)	0
5	Authenticity of the “appearance” of injuries	52 (85)	8 (13)	1 (02)
		Quite realistic	Somewhat	Not realistic
6	How realistic were the simulated injuries?	46 (75)	15 (25)	0
		Yes	May be	No
7	The injuries simulated were consistent with the scenario	60 (98)	0	19 (02)
8	The use of a moulage actor instead of a mannequin in the simulation is better	47 (77)	13 (21)	1 (02)
9	The appearance of the victim contributed positively to the training experience	60 (98)	1 (02)	0
10	Was moulage simulation excessive and distracting?	13 (21)	6 (10)	42 (69)
11	Did moulage-based simulated education help in your clinical practice?	58 (95)	2 (03)	1 (02)
12	Do you think the appearance of moulage brought on an emotional response?	32 (52)	11 (18)	18 (30)
13	This moulage-based simulation offered a good teaching-learning opportunity for the assessment and management of trauma victim	61 (100)	0	0
14	The appearance of the moulage actor made me feel like I was in a real situation	45 (74)	16 (26)	0
		Better than before	Can’t say	Worse than before
15	If you have previous experience with moulage-based simulation, how would you rate the experience in ATLS/ATCN?	35 (57)	26 (43)	0

**Table 4:** Total score of the Modified Moulage Authenticity Rating Scale (MMARS).

Maximum score = 30, Minimum score = 0	0–14 low fidelity	15–24 moderate fidelity	25–30 high fidelity
	Nil	7 (11%)	54 (89%)

## Discussion

Simulation-based trauma education has played a key role in educating medical professionals. Simulation helps to maintain the learner’s level of immersion and yields long-term knowledge retention. Simulation can use different techniques to incorporate realism. Using moulage to increase fidelity is one such technique [8-10]. Moulage was used to depict certain medical conditions with the help of a wax model. Now, moulage has transformed into special effects makeup to represent injuries and medical conditions. As simulations are expensive to perform, achieving realism can be costly. Running and maintaining a good simulation center can be costly. Using low-cost moulage can be one way to reduce the cost of running a simulation center [6,11].

The primary objective of this study was to assess the effectiveness of indigenously prepared low-cost moulage as a valid simulation tool to improve trauma education. In this study, most participants shared that the low-cost moulage had a high level of authenticity in terms of color, shape, anatomy, location, and size. Further, the general opinion of the participants was that the moulage was quite realistic. This shows that the low-cost moulage is reasonably able to mimic real injuries. This result was consistent with the results of ﻿Pywell et al. [5] who claimed that, by using low-cost materials, we can prepare high-fidelity moulage simulations.

Another significant opinion from the participants was using a moulage actor (standardized patient with moulage) was better than manikins, and the appearance of the moulage victim contributed positively to the training experience. Although we have not used manikins with moulage in our study for comparison, the participants compared it with their previous exposure to manikins in our setting. Grice et al. showed that the study participants were less satisfied with manikins and felt they could have learned better in a simulation involving standardized patients [12]. Another study by Alsaad et al. concluded that standardized patients provide live human interaction, which is a chief benefit and has shown significant improvement in learning and retention [13].

Most participants felt the moulage made the scenario so real that they felt an emotional response of urgency and got involved. Hence, they had a good learning opportunity, which will help them in their clinical practice. A study by Sezgusaya and Baskb found a similar finding: simulation with moulage effectively improved the skills in pressure injury assessment, allowing them to transfer those skills in the clinical setting [14].

Another objective of this study was to identify the pros and cons of commercial moulage items versus low-cost moulage. With previous experience using both in various educational activities, including ATLS and ATCN courses, the researchers found a few merits and demerits, which are briefly discussed in Table 5.

**Table 5:** Merits and demerits of commercial moulage versus low-cost moulage items identified by the researchers.

	Commercial moulage Items		Low-cost moulage	
	Merits	Demerits	Merits	Demerits
Availability		Only available in online stores, stocks are not always available, and many items need to be imported, leading to a delay in delivery	Readily available in the local market or daily household items can be used	
Quantity available		Only fixed quality is available	Any quantity can be availed as per need	
Cost		Very costly	Pocket friendly	
Instructions of preparing	Available online as videos and instructions			Instructions are not available. It should be made by trial and error using the imagination and creativity of the moulage
	Novice to intermediate can do moulage with these items			Needs a little skill. Only intermediate/experts can perform
Items used	Items readily available stating their purpose			Possible replacements to be identified and tested
Reuse	Often possible			Only certain items can be reused
Skin reactions	The manufacturing company claims that they are tested for manikins and human skin			Not scientifically tested, but as items are used generally in day-to-day life, it can be assumed that will not react to human skin. But the reaction to manikin should be tested before use
Duration of stay	It stays reasonably long and does not get removed/fall off easily			Short time and gets removed or loses fidelity if not handled carefully

Due to the smaller sample size and the researchers being trained on the basics of makeup and moulage from a film expert, this study is less generalizable. More studies need to be done to claim the effectiveness. A pilot study can be done to check the tool validity of the MMARS.

## Conclusions

This is possibly the first study that explores the effectiveness of low-cost moulage with high fidelity, discusses the possibilities of readily available and economic replacements in place of commercial moulage, and presents the merits and demerits of both types. The use of low-cost moulage is a feasible and economically viable method to enhance fidelity in a resource-contained setting, which can be done without the need for professional makeup artists or expensive ready-made commercial moulage kits. This technique is not limited to trauma education and can be easily translated into other simulation-based medical education domains.

## Appendices

**Table 6:** Modified Moulage Authenticity Rating Scale (MMARS).

Serial number	Topics			
	Authenticity	Good	Average	Not optimum
1	Authenticity of the color of injuries	2	1	0
2	Authenticity of the size of injuries	2	1	0
3	Authenticity of the anatomic location of injuries	2	1	0
4	Authenticity of the shape of injuries	2	1	0
5	Authenticity of the “appearance” of injuries	2	1	0
		Quite realistic	Somewhat	Not realistic
6	How realistic were the simulated injuries?	2	1	0
		Yes	May be	No
7	The injuries simulated were consistent with the scenario	2	1	0
8	The use of a moulage actor instead of a mannequin in the simulation is better	2	1	0
9	The appearance of the victim contributed positively to the training experience	2	1	0
10	Was moulage simulation excessive and distracting?	2	1	0
11	Did moulage-based simulated education help in your clinical practice?	2	1	0
12	Do you think the appearance of moulage brought an emotional response?	2	1	0
13	This moulage-based simulation offered a good teaching /learning opportunity for the assessment and management of trauma victim	2	1	0
14	The appearance of the moulage actor made me feel like I was in a real situation	2	1	0
		Better than before	Can’t say	Worse than before
15	If you have previous experience with Moulage-based simulation, how would you rate the experience in ATLS/ATCN?	2	1	0
	If you have any suggestions for improving low-cost moulage			
	Scoring and interpretation			
		High fidelity	Moderate fidelity	low fidelity
	Total score by adding each item of MMARS	21–30	11–20	0–10
